# Dynamic Alterations in Testicular Autophagy in Prepubertal Mice

**DOI:** 10.3390/jdb13040042

**Published:** 2025-11-18

**Authors:** Dong Zhang, Xiaoyun Pang, Zhenxing Yan, Weitao Dong, Zihao Fang, Jincheng Yang, Yanyan Wang, Li Xue, Jiahao Zhang, Chen Xue, Hongwei Duan, Xianghong Du, Yuxuan He

**Affiliations:** 1 College of Veterinary Medicine, Gansu Agricultural University, Lanzhou 730070, China; 13669399379@163.com (D.Z.); 17358129236@163.com (X.P.); 18394038574@163.com (Z.Y.); dongwt@gsau.edu.cn (W.D.); fangzh210@163.com (Z.F.); 1073324120274@st.gsau.edu.cn (J.Y.); 1073324120280@st.gsau.edu.cn (Y.W.); 18794864501@163.com (L.X.); 15091702209@163.com (J.Z.); lvirhgou@163.com (C.X.); duxiangh@gsau.edu.cn (X.D.); 2 College of Veterinary Medicine, Anhui Agricultural University, Hefei 230036, China; grand6138@163.com

**Keywords:** autophagy, prepubertal mice, spermatogenesis, testis

## Abstract

Autophagy has a potential regulatory effect on spermatogenesis and testicular development. Dynamic alterations in the testicular autophagy of prepubertal mice were analyzed, and the relationship between autophagy levels and testicular development was clarified using C57BL/6 mice aged 1, 2, 4, 6, and 8 weeks. Transmission electron microscopy was used to identify autophagic vacuoles. The expression of autophagy-related proteins and PI3K/AKT/mTOR signaling pathway-related proteins was determined using Western blotting. Localization of microtubule-associated protein light chain 3 (LC3) and sequestosome 1 (p62) in testicular tissues was determined using immunofluorescence and immunohistochemistry. Autophagic vacuoles in spermatogenic cells increased gradually from weeks 1 to 4, peaked at 2 weeks, decreased sharply at 6 weeks, and were undetectable at 8 weeks. The expression of Beclin 1 autophagy-related protein, LC3-II, and p62 was highest at 2 weeks among the five age groups, whereas LC3-II and p62 were mainly localized in spermatogonia and spermatocytes. Moreover, low mTOR expression and its increased expression were detected at 1–2 weeks and 2–8 weeks, respectively. These results show that testicular autophagic levels exhibit a dynamic pattern of “increase (1–2 weeks) followed by a decrease (2–8 weeks),” providing a reference in determining the relationship between autophagy levels and testicular development.

## 1. Introduction

The mammalian testis consists of seminiferous tubules, which contain spermatogenic cells, including spermatogonia, primary spermatocytes, secondary spermatocytes, and spermatids, along with Sertoli and Leydig cells [1]. The primary functions of the testes, spermatogenesis and testosterone secretion, are crucial for testicular development and male fertility [2]. Prepuberty is a critical stage in testicular development, as testicular cells undergo significant proliferation and differentiation during this period [3]. Therefore, understanding the regulatory mechanisms in testicular development before puberty is important.

Autophagy is a lysosome-mediated degradation pathway responsible for the breakdown of damaged proteins and dysfunctional organelles, allowing for the recycling of their components [4]. Intracellular autophagy is essential for maintaining cellular homeostasis under physiological conditions [5]. Increased autophagic activity can alleviate stress-induced damage caused by factors such as nutrient deficiency, hypoxia, DNA damage, and cytotoxic agents [6]. Autophagy plays a key role in preventing testicular aging [7], as insufficient autophagic activity can impair spermatogenesis and fertilization capacity [8]. The active metabolism of prepubertal testicular cells, including spermatogonia proliferation [9,10] and Sertoli cell maturation [11], generates a substantial amount of damaged components. Autophagy is therefore important for their clearance to maintain cellular homeostasis and prevent abnormal differentiation.

Dysregulated autophagy is implicated in the onset of spermatogenic disorders, which can adversely affect testicular development [12,13]. Studies have demonstrated the role of autophagy in repairing testicular damage in mice [14,15] and regulation of apoptosis during spermatogenesis [9,10,13,16,17]. Although several studies have investigated the relationship between autophagy and testicular development, little is known about the level of autophagy in the testes at different prepubertal developmental stages. Thus, autophagy levels in the testes during different stages of prepubertal development were analyzed in this study, and the underlying regulatory mechanisms were elucidated.

The PI3K/AKT/mTOR signaling pathway plays a pivotal role in regulating autophagy, with its activation generally leading to the inhibition of autophagy [18,19]. Specifically, phosphorylated PI3K activates AKT, which, in turn, phosphorylates downstream targets, ultimately suppressing autophagic activity [20]. The PI3K/AKT/mTOR pathway influences testicular development, particularly during the proliferation and differentiation of spermatogonia [9], by either inhibiting autophagy (as observed in cryptorchidism models) or regulating autophagy-associated proteins, including light chain 3 (LC3) and Beclin1. However, the direct causal relationship between this pathway and autophagy in prepubertal testicular development requires further experimental validation.

Changes in seminiferous tubules at different prepubertal developmental stages in mice were examined in this study using histological analysis. Testicular development was assessed by measuring absolute and relative weights of the testes. Moreover, the expression of autophagy marker proteins sequestosome 1 (p62) and microtubule-associated protein light chain 3 (LC3-II) in the testes at different developmental stages was analyzed. Spermatogenic cell autophagy was evaluated using transmission electron microscopy (TEM). Lastly, the expression of key proteins in the PI3K/AKT/mTOR signaling pathway was determined. Our findings provide novel insights into the dynamic changes in autophagy during prepubertal testicular development, enhancing our understanding of its regulatory mechanisms.

## 2. Materials and Methods

### 2.1. Reagents and Chemicals

LC3B polyclonal antibody (GTX127375), SQSTM1/p62 polyclonal antibody (GTX109683), and mTOR polyclonal antibody (GTX101557) were procured from GeneTex (Irvine, CA, USA). Beclin1 polyclonal antibody (AP0768), AKT polyclonal antibody (BS2987), and p-AKT polyclonal antibody (Ser473 and/or Thr308) (BS4007) were obtained from BioWorld (Dublin, OH, USA). PI3K monoclonal antibody (4257T), p-PI3K monoclonal antibody (Tyr1007/1008 of the p110 subunit) (4228T), and p-mTOR monoclonal antibody (Ser2448) (5536T) were purchased from Cell Signaling Technology (Danvers, MA, USA). β-Actin polyclonal antibody (bs-0061R) was obtained from Bioss (Beijing, China). Immunoglobulin (Ig)G (H + L) secondary antibodies (SA00001-2 and SA00001-1) and fluorescein isothiocyanate (FITC)-conjugated anti-rabbit antibody (#SA00013-2) were procured from Proteintech (Wuhan, China). The antibody diluent was TBST solution containing 5% skim milk powder.

### 2.2. Animal Experiments

All animal experiments were approved by the Animal Care Committee of Gansu Agricultural University (No. GSAU-Eth-VMC-2023-021) and conducted in accordance with the Guiding Opinions on Treating Laboratory Animals Well issued by the Ministry of Science and Technology of the People’s Republic of China.

Specific-pathogen-free-grade male C57BL/6 mice (1, 2, 4, 6, and 8 weeks of age) were purchased from the Lanzhou Institute of Veterinary Medicine, Chinese Academy of Agricultural Sciences. All mice, except 1-week-old mice, were allowed to acclimate for 1 week before experimental procedures. The mice were housed in plastic cages (29.00 × 17.80 × 16.00 cm) under controlled conditions (temperature: 23 ± 2 °C), subjected to a 12 h/12 h light/dark cycle, and provided access to deionized water and standard laboratory rodent chow ad libitum. The strain of mice used was the C57BL/6J subline introduced from the National Seed Bank, and mice used in this experiment were at the fourth passage. All mice exhibited good health status, which was verified by monitoring their food intake, water consumption, and body weight.

There were 24 mice that were 1 week old, 12 mice that were 2 weeks old, and 6 mice each that were 4, 6, and 8 weeks old. The mice were weighed and euthanized by cervical dislocation. Both testes of each mouse were immediately excised and weighed. The left testes of 3 mice from each group were placed in 4% paraformaldehyde for fixation and stored at room temperature, whereas the left testes from the other 3 mice in each group were fixed using electron microscope fixative and stored at 4 °C. The right testes of mice were stored in an ultralow-temperature refrigerator at −80 °C for backup.

### 2.3. Histological Analysis

After fixing in paraformaldehyde, testes were processed using a gradient series of ethanol and xylene, embedded in paraffin, and sectioned into 4-μm-thick slices. Hematoxylin and eosin (H&E) staining was performed, and the images were captured and analyzed using a digital microscope (Olympus, OlympusDP-71, Tokyo, Japan) at magnifications of 100× and 400×.

Ten seminiferous tubules per section were randomly selected, and their diameters were measured based on the circular tubule contour or the shortest diameter of the oval tubules. Three tissue sections from each group were analyzed as biological replicates. The 3 sections analyzed per group were derived from 3 independent mice.

### 2.4. TEM

Samples were pre-fixed in 3% glutaraldehyde, post-fixed in 1% osmium tetroxide, and dehydrated progressively using acetone. Specimens were treated with a graded mixture of a dehydrating agent and Epon-812 embedding resin before being embedded in pure Epon-812. Semi-thin sections were visualized using light microscopy (Motic, BA210 Digital, Xiamen, China), and suitable regions of the seminiferous tubules were selected to prepare ultra-thin sections (60–90 nm) using an ultramicrotome. Sections were stained with uranyl acetate and lead citrate and visualized using TEM (JEOL, JEM-1400FLASH, Tokyo, Japan).

### 2.5. Protein Extraction and Western Blotting

Total protein was extracted from testicular tissues using RIPA lysis buffer with PMSF. Sodium dodecyl sulfate–polyacrylamide gel electrophoresis (SDS-PAGE) loading buffer was added, and samples were incubated at 98 °C in a metal bath (Tiangen Biotech, OSE-DB-01, Beijing, China) and then stored at −20 °C. Proteins were separated using SDS-PAGE and transferred to a polyvinylidene fluoride membrane (Immobilon, IPVH00010, Carrigtwohill, Ireland). After blocking with 5% skim milk at room temperature for 40–60 min, the membranes were incubated overnight with the primary antibodies at 4 °C. The next day, the membranes were incubated with horseradish peroxidase-conjugated secondary antibodies at 37 °C for 1 h, and the protein bands were visualized using chemiluminescence detection (General Electric Company, Amersham Imager 600, Boston, MA, USA). Three independent replicates were performed for all Western blotting experiments, ensuring the authenticity and reliability of the results.

### 2.6. Immunofluorescence and Immunohistochemical (IHC) Assay

The localization of LC3 and p62 in testicular tissues was assessed using immunohistochemistry and immunofluorescence. Testis sections were deparaffinized, rehydrated, and subjected to antigen retrieval using 0.01 M sodium citrate buffer (pH 6.0). Endogenous peroxidase activity was blocked using 3% hydrogen peroxide for 5–10 min. After blocking with goat serum for 20 min at room temperature, the sections were incubated overnight with an anti-LC3 antibody (1:200 dilution) at 4 °C. The sections were rewarmed, incubated with anti-rabbit IgG for 1 h at room temperature, and developed using a diaminobenzidine staining kit. Lastly, the tissue sections were counterstained with hematoxylin and then dehydrated, transparentized, and mounted.

The procedure for the immunofluorescence assay was similar to that for the IHC assay, except that the sections were incubated with an FITC-conjugated anti-rabbit antibody (1:600 dilution) instead of a peroxidase-conjugated secondary antibody. Fluorescence microscopy (Olympus, LX71-DP71, Japan) was used to visualize protein expression [21,22,23]. All analyses were based on 3 biological replicates (sections from three independent mice per group) to ensure reliability.

### 2.7. Statistical Analysis

Data were processed and analyzed using ImageJ 1.44p Java 1.6.0-20 (32bit) (for quantitative analysis of experimental images, National Institutes of Health, Bethesda, MD, USA), Origin Pro 9 (for statistical calculations, OriginLab Corporation, Northampton, MA, USA), and GraphPad Prism 8.0.2 (for data plotting, GraphPad Software, Inc., San Diego, CA, USA). Protein expression levels were quantified as grayscale values, and the protein bands were scanned and analyzed using ImageJ. Similarly, the positive immunofluorescence signals were analyzed using ImageJ. Continuous variables, including body weight and testis weight, are presented as mean ± standard deviation (SD). Group differences in these variables were assessed using one-way analysis of variance (ANOVA). *p* < 0.05 was considered statistically significant.

## 3. Results

### 3.1. Body Weight, Testis Weight, and Relative Testis Weight in Prepubertal Mice

**Figure 1:**
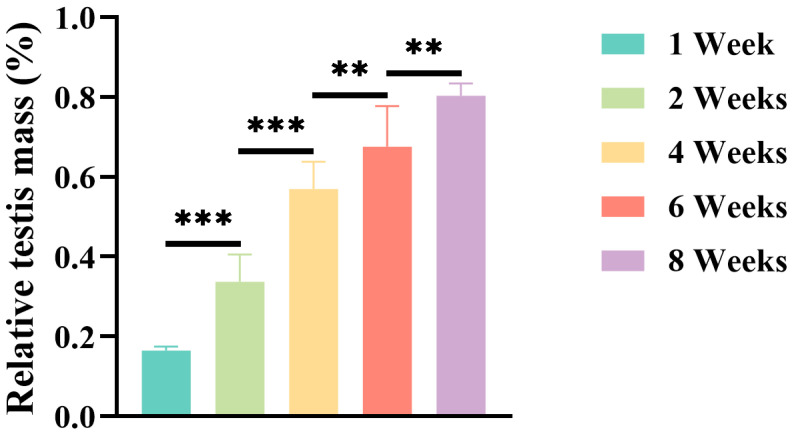
Age-dependent changes in relative testis weight during prepubertal development of mice. Data are presented as mean ± SD (*n* = 6 per group). Relative testis weight was calculated as (testes weight/body weight) × 100%. Statistical significance was determined using one-way ANOVA with Tukey’s post hoc test for sequential pairwise comparisons(** *p* < 0.01, *** *p* < 0.001 vs. the preceding age group).

The body weight and testis weights of mice at different stages of prepubertal development are presented in Table 1, whereas the relative testis weight is illustrated in Figure 1. Both absolute and relative testis weights increased significantly with an increase in age.

The testis weight and relative testis weight of 2-, 4-, 6-, and 8-week-old mice were significantly higher (*p* < 0.001) compared with those in 1-week-old mice. Similarly, the testis weight and relative testis weight of 4-, 6-, and 8-week-old mice exhibited a significant increase (*p* < 0.001) compared with those in 2-week-old mice. Moreover, the testis weight and relative testis weight of 6- and 8-week-old mice were also significantly higher (*p* < 0.01) compared with those of 4-week-old mice. Lastly, both testis weight and relative testis weight of 8-week-old mice showed a significant increase (*p* < 0.01) compared with those of 6-week-old mice.

### 3.2. Histological Changes in Testicular Development

**Figure 2:**
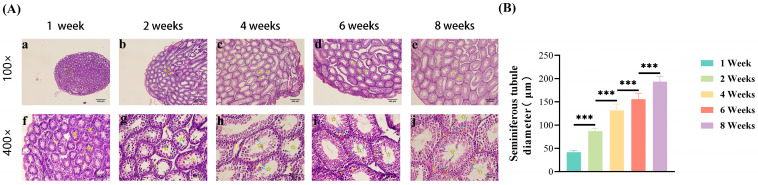
Microstructural and morphometric changes in the testes of prepubertal mice. (**A**) Developmental progression of seminiferous tubules. Representative H&E staining sections at 100× (**a**–**e**; scale bar = 200 μm) and 400× magnification (**f**–**j**; scale bar = 50 μm). Key structures: seminiferous tubules (★), spermatogonia (↑), primary spermatocytes (↑), secondary spermatocytes (↑), spermatids (↑), spermatozoa (↑), Sertoli cells (↑), and Leydig cells (↑). Developmental stages correspond to the emergence and maturation of germ cell types described in the results section. (**B**) Sequential expansion of seminiferous tubule diameter during prepubertal development. Tubule diameters were quantified using ImageJ. Data are presented as mean ± SD (*n* = 30 tubules per group). Statistical significance was determined using one-way ANOVA with Tukey’s post hoc test for sequential pairwise comparisons between adjacent age groups: *** *p* < 0.001 (2 weeks vs. 1 week; 4 weeks vs. 2 weeks; 6 weeks vs. 4 weeks; 8 weeks vs. 6 weeks).

Figure 2A illustrates microstructural changes in the testes of mice at different prepubertal developmental stages, as identified using H&E staining. At 1 week of age, the population of testicular cells primarily consisted of spermatogonia, primary spermatocytes, Sertoli cells, and Leydig cells. Secondary spermatocytes emerged at 2 weeks of age, whereas spermatids became apparent at 4 weeks of age. The number of spermatocytes increased significantly at 6 weeks of age, and spermatozoa were observed. At 8 weeks of age, the spermatozoa were clustered together within seminiferous tubules, indicating ongoing spermatogenesis.

### 3.3. Changes in Seminiferous Tubule Diameter

Figure 2B demonstrates age-related changes in the diameter of seminiferous tubules. A significant increase in tubule diameter was observed with advancing age. Seminiferous tubule diameters of 2-, 4-, 6-, and 8-week-old mice were significantly larger (*p* < 0.01) compared with those of 1-week-old mice. Similarly, the tubule diameters of 4-, 6-, and 8-week-old mice exhibited a significant increase (*p* < 0.01) compared with those of 2-week-old mice. Furthermore, the tubule diameters of 6- and 8-week-old mice were significantly increased (*p* < 0.01) compared with those of 4-week-old mice. Lastly, the tubule diameters of 8-week-old mice showed a further significant increase compared with those of 6-week-old mice (*p* < 0.01).

These findings indicate that all major types of spermatogenic cells progressively populate the seminiferous tubules before puberty. Additionally, the diameter of these tubules increased substantially during testicular development, further supporting the maturation of the testicular structure.

### 3.4. Observation of Autophagy

**Figure 3:**
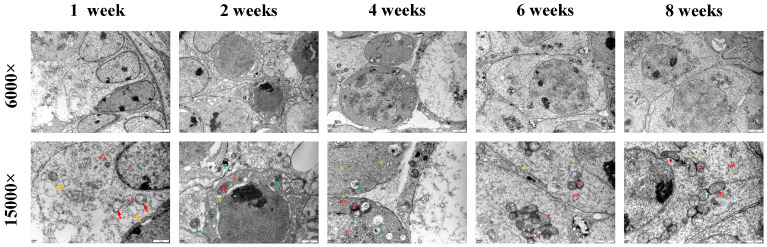
Prepubertal testicular ultrastructure reveals age-dependent autophagy dynamics. Transmission electron micrographs of seminiferous tubules at 6000× (scale bar = 2 μm) and 15,000× (scale bar = 500 nm) showing the nucleus (N), mitochondria (Mi), rough endoplasmic reticulum (RER) (↑), swollen mitochondria (↑), and autophagosomes (↑).

TEM revealed distinct developmental changes in the seminiferous tubules and mitochondrial morphology across mice of various age groups. At 1 week of age, the seminiferous tubules were not completely developed, and the mitochondria in spermatocytes appeared swollen, without evidence of autophagy. At 2 weeks of age, the seminiferous tubules remained underdeveloped; however, the mitochondria in both spermatocytes and spermatogonia exhibited swelling, and autophagic activity had noticeably increased. At 4 weeks of age, the seminiferous tubules had developed completely, and the mitochondria in the spermatogonia and spermatocytes exhibited only mild swelling, along with significant autophagy. At 6 weeks of age, the seminiferous tubules remained fully developed. The mitochondria in spermatocytes, spermatogonia, and spermatids appeared either mildly swollen or normal, whereas autophagic activity was reduced. At 8 weeks of age, the seminiferous tubules were completely developed, mitochondria in the spermatogenic cells (spermatocytes, spermatogonia, and spermatids) appeared normal, and no autophagy was detected (Figure 3). Overall, the mitochondrial morphology and structure were found to be increasingly intact in spermatogenic cells as the mice matured, with relatively high levels of autophagic activity observed at 2 and 4 weeks of age.

### 3.5. Expression of LC3 and Beclin1 During Testicular Development

LC3 and Beclin1 are key marker proteins indicative of the extent of autophagy in the testes. To assess autophagic activity during prepubertal testicular development, the expression of LC3 and Beclin1 was analyzed using Western blotting.

**Figure 4:**
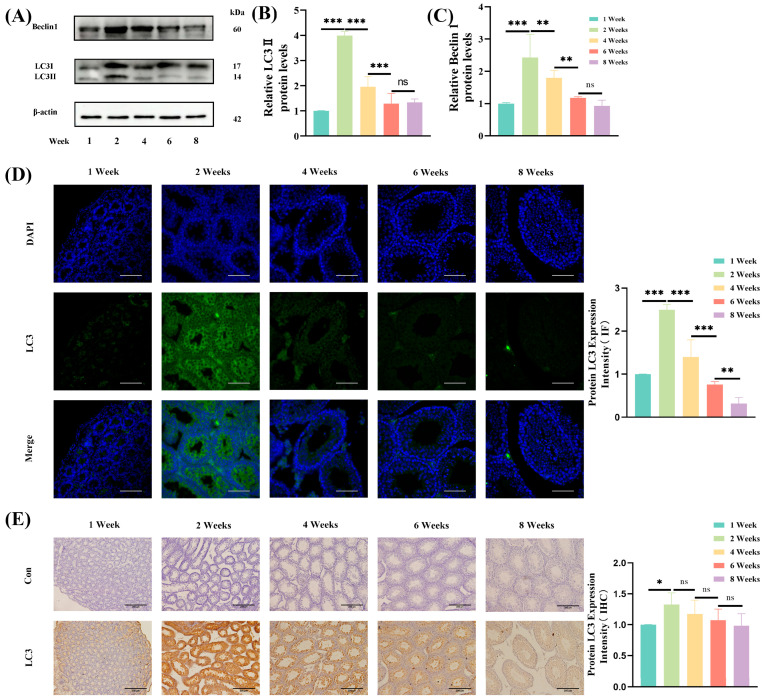
Expression of autophagy marker proteins in the testes of prepubertal mice. (**A**) Western blotting to determine Beclin1 and LC3 expression levels in the testes of mice of different ages (1, 2, 4, 6, and 8 weeks). (**B**) Quantitative analysis of relative LC3-II protein levels across developmental stages. (**C**) Quantitative analysis of relative Beclin1 protein levels across developmental stages. (**D**) Immunofluorescence (IF) staining showing LC3 expression (green) and nuclear counterstaining with DAPI (blue) in seminiferous tubules at different developmental stages. 20×, scale bar = 200 μm. (**E**) Immunohistochemical (IHC) analysis of LC3 expression (brown) in the seminiferous tubules at different developmental stages, with negative controls showing minimal background staining. 20×, scale bar = 200 μm. * *p* < 0.05, ** *p* < 0.01, *** *p* < 0.001, ns: not significant.

LC3 expression increased significantly (*p* < 0.001) with an increase in age from 1 weekto 2 weeks and peaked at 2 weeks of age. LC3 expression exhibited a subsequent significant decline (*p* < 0.001) from 2 weeks to 6 weeks of age, and without any significant difference in expression in mice between 6 weeks and 8 weeks of age (Figure 4A,B). Similarly, Beclin1 expression increased significantly from 1 week to 2 weeks of age (*p* < 0.001), peaking at 2 weeks of age and then exhibiting a marked decline from 2 weeks to 6 weeks of age (*p* < 0.01). No significant difference in Beclin1 expression was noted between mice that were 6 weeks to 8 weeks of age (Figure 4A,C).

Immunofluorescence analysis demonstrated a significant increase in LC3 expression from 1 week to 2 weeks of age (*p* < 0.001), reaching a maximum at 2 weeks, and then exhibiting a significant decrease from 2 weeks to 8 weeks of age (*p* < 0.01) (Figure 4D), further confirming these findings. These findings were in agreement with those from Western blotting. Similarly, immunohistochemical analysis showed a significant increase in LC3 expression from 1 week to 2 weeks of age (*p* < 0.05), peaking at 2 weeks, and then declining from 2 weeks to 8 weeks of age (Figure 4E). These results were consistent with the findings from Western blotting and immunofluorescence analysis.

### 3.6. p62 Expression During Testicular Development

**Figure 5:**
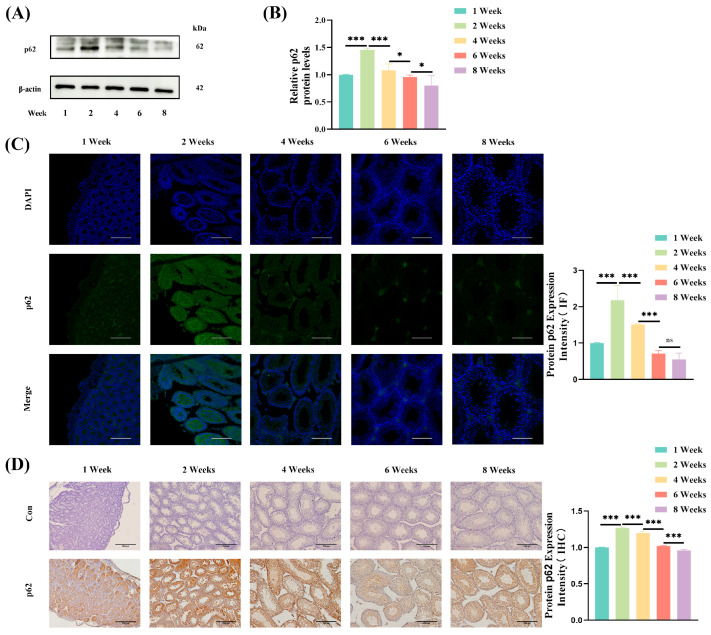
Expression of the autophagy marker protein p62 in the testes of prepubertal mice. (**A**) Western blotting to determine p62 expression in the testes of mice at different developmental stages (1, 2, 4, 6, and 8 weeks). (**B**) Quantitative analysis of relative p62 protein levels across developmental stages. (**C**) Immunofluorescence staining showing p62 expression (green) and nuclear counterstaining with DAPI (blue) in the seminiferous tubules of mice at different developmental stages. 20×, scale bar = 200 μm. (**D**) Immunohistochemical (IHC) analysis of p62 expression (brown) in the seminiferous tubules at different developmental stages of mice, with negative controls showing minimal background staining. 20×, scale bar = 200 μm. * *p* < 0.05, *** *p* < 0.001. ns: not significant.

p62 is a well-established marker protein that is indicative of the extent of autophagy in the testes. To evaluate the dynamics of autophagy during prepubertal testicular development, p62 expression was analyzed using Western blotting. The expression of p62 increased significantly (*p* < 0.001) with increasing age, from week 1 to 2 weeks, and peaked at 2 weeks of age. Subsequently, p62 expression decreased significantly (*p* < 0.05) from 2 weeks to 8 weeks of age (Figure 5A,B). Immunofluorescence analysis further confirmed these findings, indicating a significant increase in p62 expression from 1 week to 2 weeks of age (*p* < 0.001) and attaining the highest level at 2 weeks of age, and then declining markedly from 2 weeks to 6 weeks of age (*p* < 0.001) (Figure 5C). These results were consistent with the findings from Western blotting.Similarly, IHC analysis demonstrated a significant increase in p62 expression from 1 week to 2 weeks of age (*p* < 0.001), peaking at 2 weeks of age, and then exhibiting a decreasing trend from 2 weeks to 8 weeks of age (*p* < 0.001) (Figure 5D). These findings align with the results from Western blotting and immunofluorescence.

### 3.7. Autophagy Pathway

**Figure 6:**
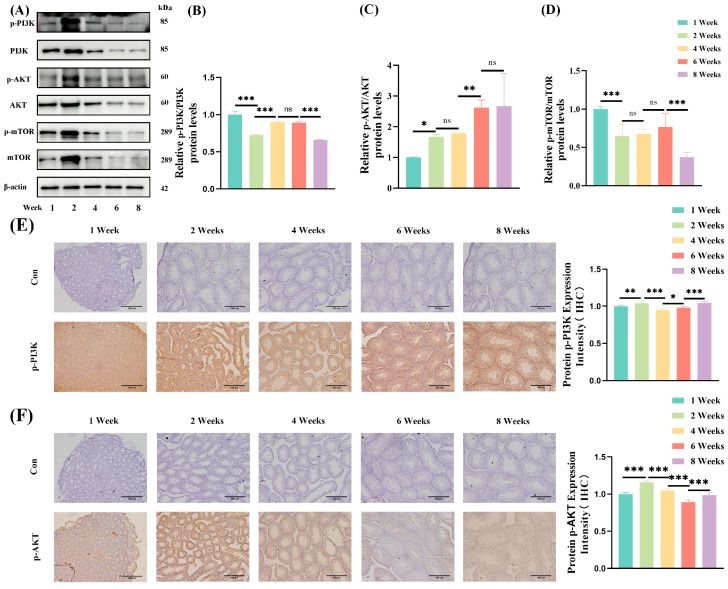
Expression of autophagy pathway-related proteins in the testes of prepubertal mice. (**A**) Western blotting demonstrating the expression of PI3K, p-PI3K, AKT, p-AKT, mTOR, and p-mTOR proteins at different developmental stages (1, 2, 4, 6, and 8 weeks) of mice. (**B**) Quantitative analysis of relative p-PI3K/PI3K protein levels across developmental stages of mice. (**C**) Quantitative analysis of relative p-AKT/AKT protein levels across developmental stages of mice. (**D**) Quantitative analysis of relative p-mTOR/mTOR protein levels across developmental stages of mice. (**E**) Immunohistochemical (IHC) analysis of p-PI3K expression (brown) in the seminiferous tubules at different developmental stages of mice, with negative controls showing minimal background staining. 20×, scale bar = 200 μm. (**F**) Immunohistochemical (IHC) analysis of p-AKT expression (brown) in the seminiferous tubules at different developmental stages of mice, with negative controls showing minimal background staining. 20×, scale bar = 200 μm. * *p* < 0.05, ** *p* < 0.01, *** *p* < 0.001, ns: not significant.

The PI3K/AKT/mTOR pathway plays a pivotal role in regulating autophagy in the testes of mice. To investigate potential changes in this pathway during preadult development, the protein expression of PI3K, AKT, and mTOR, along with their phosphorylation states, was quantified using protein blotting techniques. A dynamic pattern of PI3K expression was noted across developmental stages. Specifically, the p-PI3K/PI3K ratio was significantly reduced from 1 week to 2 weeks of age (*p* < 0.001) and then demonstrated an ascending trend until 6 weeks of age (*p* < 0.001), followed by a subsequent significant decrease from 6 weeks to 8 weeks of age (*p* < 0.001). No significant variations were observed between 4 weeks and 6 weeks of age (Figure 6A,B). The phosphorylation ratio of AKT (p-AKT/AKT) demonstrated an overall upward trajectory. Significant increases were noted between 1 week and 2 weeks (*p* < 0.05) and between 4 weeks and 6 weeks of age (*p* < 0.01) (Figure 6A,C). Conversely, the p-mTOR/mTOR ratio was significantly reduced from 1 week to 2 weeks of age (*p* < 0.01), and then demonstrated an ascending trend until 6 weeks of age, followed by a subsequent significant decrease from 6 weeks to 8 weeks of age (*p* < 0.01) (Figure 6A,D). These findings were corroborated by the findings from IHC analyses, indicating a substantial elevation in p-PI3K expression from 1 week to 2 weeks of age (*p* < 0.01), a decline from 2 weeks to 4 weeks of age (*p* < 0.001), and a resurgence from 4 weeks to 8 weeks of age (*p* < 0.05) (Figure 6E). Similarly, p-AKT expression showed a significant surge from 1 week to 2 weeks of age (*p* < 0.001), peaked at 2 weeks of age, and then gradually diminished until 6 weeksof age (*p* < 0.001) (Figure 6F), aligning with the findings from Western blotting.

## 4. Discussion

The dynamic changes in autophagy in the testes of prepubertal mice at different developmental stages were determined in this study.A significant increase in both absolute and relative testis weights, as well as in the diameter of seminiferous tubules, was noted as mice progressed toward adulthood. Histological analyses further demonstrated a marked increase in the number and diversity of spermatogenic cells in the seminiferous tubules. These findings collectively suggest that spermatogenic cells undergo rapid proliferation in the testes of mice before puberty, consistent with the establishment of functional spermatogenesis. This result is consistent with the findings of a previous study [24].

The relationship between testicular development and autophagy involves complex physiological interactions that affect key structural and functional parameters. Autophagy plays a crucial role in maintaining testicular homeostasis, with alterations leading to measurable changes in testicular morphology [25,26]. Disturbances in autophagy have been associated with increased seminiferous tubule diameter [25], whereas proper autophagy regulation helps preserve the integrity of the seminiferous tubules [26]. Physiological parameters such as testis weight show dose-dependent reductions when autophagy is dysregulated [14], although some studies report minimal impact on absolute testicular weight under certain conditions [27]. The diameter of seminiferous tubules is a particularly sensitive indicator, with multiple studies reporting alterations in response to autophagy modulation [28,29]. These morphological changes are often correlated with functional impairments, as evidenced by decreased epithelial thickness [30] and reduced spermatogenic cell populations [31]. The interaction between autophagy and testicular development appears bidirectional, where both excessive autophagy induction [28] and impaired autophagic flux [14] can disrupt the delicate balance required for normal spermatogenesis and maintaining the testicular architecture.

TEM was used to investigate age-related changes in testicular autophagy. Improvements in mitochondrial morphology and structural integrity in germ cells were noted. Notably, autophagy levels peaked at 2 weeks and 4 weeks of age, followed by a decline.Although our study did not elucidate the role of mitochondrial autophagy, TEM revealed mitochondrial changes, suggesting the involvement of mitochondrial autophagy in regulating testicular development.Mitophagy plays a crucial role in the development of the testes by maintaining mitochondrial homeostasis and supporting spermatogenesis [32]. Studies have demonstrated that SPATA33 mediates mitophagy in testes by binding to VDAC2 on mitochondria, facilitating autophagic clearance during starvation [33]. This finding confirms our hypothesis that mitochondrial autophagy is involved in regulating testicular development. In future studies, we intend to determine the levels of the mitochondrial autophagy marker proteins PINK1, Parkin, BNIP3, and NIX; elucidate the relationship between mitochondrial autophagy and testicular development; and supplement and improve the mechanism of autophagy regulation of testicular development.

Autophagy, a conserved process of intracellular degradation, is regulated by key markers such as LC3 and p62 [34]. Our findings indicated that autophagy levels in the testes of mice increased from 1 week to 2 weeks of age and subsequently decreased from 2 weeks to 8 weeks of age. LC3, a protein essential for autophagosome formation, exists in two forms, namely, cytoplasmic LC3-I and membrane-bound LC3-II. The conversion of LC3-I to LC3-II is a hallmark of autophagy initiation. Similarly, p62 (SQSTM1), a substrate-binding protein, is degraded during functional autophagy, and its accumulation is indicative of impaired autophagic flux [35]. From 1 week to 2 weeks of age, the increase in LC3-II and Beclin1 was suggestive of enhanced autophagy initiation, whereas the concurrent high level of p62 indicates possible impairment in downstream autophagic flux (i.e., incomplete clearance of autophagosomes), likely due to defective fusion of the autophagosomes with lysosomes. This blockage resulted in the accumulation of undegraded p62 [36]. In contrast, the simultaneous decrease in LC3 and p62 levels from 2 weeks to 8 weeks of age indicated unimpeded autophagic flux and normal degradation of p62, signifying functional autophagy during this period.Our findings are supported by those reported previously, which confirm that coordinated changes in LC3, Beclin1, and p62 are key indicators of autophagic flux balance. For example, Tat-Beclin1–stimulated models showed increased Beclin1, LC3-II, and p62, mimicking our 1–2-week-old “enhanced initiation with p62 accumulation [37]“, whereas salvianin B in myocardial ischemia upregulated LC3/Atg5/Beclin1 and reduced p62, consistent with our 2–8-week-old normal autophagy [38]. Pathological studies have further validated that nerve injury causes sustained LC3-II increase and a 3-fold accumulation of p62 (alleviated by Beclin-1 F121A) [39], and that cadmium-exposed Sertoli cells show excessive ATG7/Beclin1/LC3/p62 activation [40]. Collectively, LC3/Beclin1/p62 co-expression accurately reflects the balance in autophagosome formation and degradation, distinguishing between physiological and pathological autophagic states. Future studies should link upstream/downstream pathways (e.g., mTOR/AMPK) to clarify the regulatory mechanisms for targeted autophagy intervention [41,42].

The changes in autophagy levels that were observed may be linked to key developmental events such as testicular descent, which typically occurs between the second and fifth weeks of life in mice [43]. At 2 weeks of age, ongoing gonadal development and fluctuations in the levels of sex hormones may upregulate autophagy-related genes, enhancing autophagic activity [17]. Furthermore, the decline in prostaglandin (PG) levels from 2 weeks to 8 weeks of age, coinciding with the onset of puberty and increased testosterone production, may further modulate autophagy [44]. Testosterone modulates autophagy via multiple pathways––it enhances autophagy induced by amyloid-beta (Aβ) in the microglia by suppressing the phosphorylation of extracellular signal-regulated kinase and activation of mammalian target of rapamycin, thereby promoting the clearance of Aβ. This may reduce the susceptibility of males to Alzheimer’s disease [45]. In Leydig cells, autophagy flux regulates testosterone biosynthesis via the METTL3–m6A–SIRT1 axis, with both excessive and deficient autophagy impairing testosterone production [46]. Although PGs have not been directly studied in regulating autophagy in the cited literature, they are involved in immunomodulation [47], which may interact with the autophagy pathway, particularly in inflammatory contexts, where they coordinate with cytokines and matrix metalloproteases [48]. The relationship between these hormones and autophagy is cell-type specific, with testosterone demonstrating dual roles in either promoting [45] or inhibiting [46] autophagy depending on the cellular context and pathological conditions. These findings suggest the significant role of hormonal changes during sexual maturation in regulating autophagy in the testis.

The regulation of autophagy during testicular development is intricately linked to the PI3K/AKT/mTOR signaling pathway across multiple stages [49]. Autophagy-related genes participate in regulatory processes within the hypothalamic–pituitary–testis axis, whereas mTORC1 (a key autophagy gatekeeper) is crucial for spermatogonial stem cell proliferation, meiotic progression, and spermiogenesis [9]. The PI3K/AKT/mTOR pathway is explicitly implicated in these processes, as evidenced by upregulated pathway activity in gonadal tissues and its modulation by retinoic acid to restore the function of the blood–testis barrier via RARα [50,51]. Activation of PI3K by extracellular or intracellular signals leads to the production of phosphatidylinositol trisphosphate, which activates AKT through phosphatidylinositol-dependent kinase 1. Activated AKT (phosphorylated AKT) subsequently stimulates mTOR, promoting cell growth and protein synthesis while inhibiting autophagy [52]. Our findings demonstrated that low p-AKT levels from 1 week to 2 weeks of age resulted in the inhibition of mTOR and increased autophagy. Conversely, increased p-AKT levels from 2 weeks to 6 weeks of age activated mTOR, leading to a decline in autophagy [53]. These findings align with the observed trends in autophagy-related protein expression, confirming the regulatory role of this pathway. Our study primarily reveals a correlation between PI3K/AKT/mTOR pathway activity and autophagy changes. Functional experiments (e.g., pathway inhibition using PI3K/AKT/mTOR inhibitors [54,55,56], mTOR activation using specific activators [57,58,59]) in future studies will help verify the causal regulatory role of this pathway.

## 5. Conclusions

Correlative evidence regarding dynamic alterations of autophagy in the testes of prepubertal mice across distinct developmental stages is presented in this study. From 1 week to 4 weeks of age, the number of autophagic vacuoles in spermatogenic cells depicted a gradual upward trend, with a peak observed at 2 weeks. This number then dropped sharply by 6 weeks and was undetectable at 8 weeks. Among the five age groups that were analyzed, the expression levels of autophagy-related proteins Beclin1, LC3-II, and p62 attained the highest levels at 2 weeks. Further localization analysis indicated LC3-II and p62 to be predominantly distributed in spermatogonia and spermatocytes. Moreover, mTOR expression was low during the 1–2 week period; it started to increase after 2 weeks and continued to increase until 8 weeks. These findings collectively demonstrate that autophagic activity in the testes of prepubertal mice follows a dynamic trajectory of “elevation (1–2 weeks) and subsequent reduction (2–8 weeks),” thereby serving as a foundational reference in determining the association between testicular autophagy levels and testicular developmental processes.

## Figures and Tables

**Table 1:** Age-dependent changes in the body weight and testis weight of prepubertal mice.

Age	Body Weight (g)	Testes Weight (g)
1 week	2.62 ± 0.49	0.0043 ± 0.0010
2 weeks	5.63 ± 0.74	0.0190 ± 0.0073
4 weeks	9.12 ± 1.43	0.0520 ± 0.0101
6 weeks	15.81 ± 1.36	0.1072 ± 0.0213
8 weeks	18.64 ± 0.90	0.1495 ± 0.0056

Data are presented as mean ± SD (*n* = 6 per group).

## Data Availability

All the data are presented in the manuscript.
